# Deep targeted sequencing of circulating tumor DNA to inform treatment in patients with metastatic castration-resistant prostate cancer

**DOI:** 10.1186/s13046-025-03356-0

**Published:** 2025-04-14

**Authors:** Maibritt Nørgaard, Maria Rusan, Karoline Kondrup, Ea Marie Givskov Sørensen, Simone Weiss, Marianne Trier Bjerre, Jacob Fredsøe, Søren Vang, Jørgen Bjerggaard Jensen, Bram De Laere, Henrik Grönberg, Michael Borre, Johan Lindberg, Karina Dalsgaard Sørensen

**Affiliations:** 1 Present Address: Department of Molecular Medicine, Aarhus University Hospital, Palle Juul-Jensens Boulevard 99, Aarhus, 8200 Denmark; 2 Department of Clinical Medicine, Aarhus University, Aarhus, Denmark; 3 Present Address: Department of Clinical Pharmacology, Aarhus University Hospital, Aarhus, Denmark; 4 Department of Urology, Aarhus University Hospital, Aarhus, Denmark; 5 Department of Urology, Gødstrup Hospital, Gødstrup, Denmark; 6 Department of Medical Epidemiology and Biostatistics, Karolinska Institutet, Stockholm, Sweden; 7 Department of Human Structure and Repair, Ghent University, Ghent, Belgium; 8 Cancer Research Institute Gent (CRIG), Ghent University, Ghent, Belgium

**Keywords:** Metastatic castration-resistant prostate cancer (mCRPC), Circulating cell-free DNA (cfDNA), Circulating tumor DNA (ctDNA), Liquid biopsy, Biomarker, Prognostic, Predictive, Resistance, Clinical actionability, Clonal hematopoiesis

## Abstract

**Background:**

Intrinsic and acquired resistance to second-generation anti-androgens pose a significant clinical challenge in the treatment of metastatic castration-resistant prostate cancer (mCRPC). Novel biomarkers to predict treatment response and inform alternative treatment options are urgently needed.

**Methods:**

Deep targeted sequencing, with a prostate cancer-specific gene panel, was performed on circulating tumor DNA (ctDNA) and germline DNA from blood of mCRPC patients recruited in Denmark (n = 53), prior to starting first-line treatment with enzalutamide or abiraterone acetate, and for a subset of patients also at progression (n = 18). Likely clonal hematopoietic variants were filtered out. Genomic findings were correlated to clinical outcomes (PSA progression-free survival (PFS), overall survival (OS)). Intrinsic resistance candidate biomarkers were considered by enrichment analysis of nonresponders vs. responders. Genomic alterations at progression were considered as possible drivers of acquired resistance. Clinical actionability was assessed based on OncoKB and ESCAT.

**Results:**

Somatic alterations in *PTEN*, cell cycle regulators (*CCND1, CDKN1B, CDKN2A*, and *RB1*) and chromatin modulators (*CHD1, ARID1A*) were associated with significantly shorter PFS and OS, also after adjusting for ctDNA% in multivariate Cox regression analysis. The associations with poorer outcomes for alterations in *PTEN* and chromatin modulators were validated in an external dataset. Patients with primary resistance to enzalutamide/abiraterone had enrichment for *BRAF* amplification and *CHD1* loss, while responders had enrichment for *TMPRSS2* fusions. *AR* resistance mutations emerged in 22% of patients at progression. These were mutually exclusive with other alterations that may confer resistance (i.e., activating *CTNNB1* mutations, combined *TP53/RB1* loss). Clinically actionable alterations, primarily in homologous recombination repair genes, were found in 54.7% and 49.0% of patients (OncoKB and ESCAT, respectively), with few additional alterations detected at progression. Level I alterations were identified in 41.5% of patients employing OncoKB, however only in 13.2% based on ESCAT.

**Conclusions:**

Our study identifies known and novel prognostic and predictive biomarker candidates in patients with mCRPC undergoing first-line treatment with enzalutamide or abiraterone acetate. It further provides real-world evidence of the significant potential of genomic profiling of ctDNA to inform treatment in this setting. Clinical trials are warranted to advance the implementation of ctDNA-based biomarkers into clinical practice.

**Supplementary Information:**

The online version contains supplementary material available at 10.1186/s13046-025-03356-0.

## Background

Life-prolonging treatment options for metastatic castration-resistant prostate cancer (mCRPC) have rapidly expanded in recent years, including the approval of second-generation anti-androgens (i.e., enzalutamide and abiraterone acetate). Despite these advancements, major clinical challenges remain, with a significant proportion of patients presenting with primary resistance to these agents (c.10–40%) and acquired resistance inevitably developing in the remaining patients [1–3]. Novel biomarkers that can predict treatment response, and inform alternative treatments, are thus urgently needed to guide treatment selection in this patient population.

Unlike in other cancer types, genomic profiling of tumor tissue is not routinely employed in clinical practice for patients with mCRPC. This is in part due to tumor tissue not being easily accessible, as patients often solely have bone metastases, and in part due to the conceived limited clinical utility of tumor profiling in this patient population. Nevertheless, recent studies characterizing the molecular landscape of mCRPC have identified androgen receptor (AR) splice variants, *AR* mutations and copy number gains, as well as activation of alternative pathways (e.g., WNT or FGFR signaling), as mechanisms conferring resistance to second-generation anti-androgens [3–7]. Moreover, PARP inhibitors have recently been approved as monotherapy, as well as in combination with second-generation anti-androgens [8–10], for patients with mCRPC with either germline or somatic alterations in homologous recombination repair genes (e.g., *BRCA2)*. These developments suggest a clinically meaningful role for genomic profiling in this patient population and warrant further investigation into genomic biomarkers to inform treatment in this setting.

Profiling of circulating tumor DNA (ctDNA) in blood has been suggested to be advantageous in this patient population, as it is minimally invasive, able to identify the genomic driver alterations present in matched prostatic tumor tissue [11, 12], and as it may more closely capture the heterogeneity of the metastatic burden in each patient [12]. In this study, we therefore sought to identify ctDNA-based biomarkers associated with treatment response, progression and survival in patients with mCRPC undergoing first-line treatment with enzalutamide or abiraterone acetate. We furthermore sought to investigate the utility of ctDNA profiling to identify clinically actionable alterations that may inform targeted therapy options. Accordingly, we performed deep targeted sequencing of 71 liquid biopsies from 53 men with newly diagnosed mCRPC, employing a comprehensive prostate cancer (PC)-tailored gene panel, enabling identification of mutations in 78 relevant genes and structural variants in 11 genes, as well as estimation of copy number and determination of microsatellite instability (MSI) status [13–15]. ctDNA from plasma, and germline DNA from buffy coats, were sequenced at baseline (mCRPC diagnosis, prior to commencement of first-line treatment), and for a subset of patients also at progression (18/53 patients, taken at time of treatment cessation).

Our study builds and expands on prior studies, and provides real-world evidence of the significant potential of genomic profiling of ctDNA to inform treatment in patients undergoing first-line treatment for mCRPC.

## Patients and methods

### mCRPC patients

Blood samples (30–50 mL) were obtained from 53 mCRPC patients, receiving abiraterone acetate or enzalutamide as first-line treatment, at Aarhus University Hospital or the Regional Hospital West Jutland, Denmark (Table 1). Patients were selected, based on having ctDNA% estimates of ≥ 3% using ichorCNA [16], from a larger cohort of mCRPC patients previously profiled by low-pass whole genome sequencing (lpWGS) [17]. Patients were included between April 1st, 2016 and August 31st, 2018. Samples were collected at mCRPC diagnosis prior to treatment initiation (*n* = 53) and for a subset also longitudinally during treatment (median interval of 2 months, range 0.5–5.6 months) and at progression when treatment was discontinued (*n* = 18).

**Table 1:** Patient characteristics

Patient characteristics	Cohort (n = 53)
**M-stage at initial PC diagnosis, n (%)**	
0	23 (43.4)
1	28 (52.8)
X	2 (3.8)
**Treatment with curative intent at initial PC diagnosis, n (%)**	
Radical prostatectomy	5 (9.4)
Radiation therapy	9 (17.0)
Not treated with curative intent	39 (73.6)
**Treatment in the hormone-sensitive setting, n (%)**	
ADT alone	38 (71.7)
Surgical castration alone	4 (7.5)
ADT + surgical castration	1 (1.9)
ADT + docetaxel	10 (18.9)
**Metastatic burden at mCRPC diagnosis (imaging), n (%)**	
Bone only	23 (43.5)
Lymph node only	6 (11.3)
Bone and lymph node	19 (35.8)
Visceral	5 (9.4)
**Blood chemistry at mCRPC diagnosis (baseline)**	
PSA (ng/mL), median (range)	46.3 (1.4–350.6)
Alkaline phosphatase (U/L), median (range)	111 (9.6–1053)
**ECOG Performance status at mCRPC diagnosis, n (%)**	
0	28 (52.9)
1	21 (39.6)
2	4 (7.5)
**Overall follow-up (months), median (range)**	22.2 (2.6–57.9)
**First-line mCRPC treatment, n (%)**	
Abiraterone acetate	15 (28.3)
Enzalutamide	38 (71.7)
**PSA progression, first-line mCRPC treatment**	
Yes, n (%)^a^	47 (88.7)
Progression-free survival (months), median (range)	6.9 (1.3–28.0)
No, n (%)^b^	6 (11.3)
Available follow-up time during first-line treatment (months), median (range)	14.5 (1.0–48.6)
**PSA response, first-line mCRPC treatment**	
PSA30, n (%)^c^	45 (86.5%)
PSA50, n (%)^c^	45 (86.5%)
PSA90, n (%)^c^	28 (53.8%)
**Dead**	
Yes, n (%)	37 (69.8)
Overall survival (months), median (range)	22.2 (3.6–49.7)
No, n (%)	16 (30.2)
Total available follow-up time (months), median (range)	21.5 (2.6–57.9)

*PC* prostate cancer, *ADT* androgen deprivation therapy, *mCRPC* metastatic castration-resistant prostate cancer, *PSA* prostate specific antigen

^a ^Subsequently confirmed by imaging (radiographic progression) in 47/47 (100%) patients

^b ^Three of the 6 patients passed away prior to PSA progression, one patient was lost to follow-up, one was switched from abiraterone to enzalutamide after one month of treatment due to side effects rather than progression, and one was still on treatment at last follow-up

^c ^One patient switched from abiraterone to enzalutamide after one month of treatment due to side effects and was therefore not included in this analysis

### Blood sample processing and extraction of circulating cell-free DNA (cfDNA) and germline DNA

Blood samples were collected in 10 mL BD Vacutainer K_2_ EDTA tubes (Beckton Dickinson) and processed within 2 h as previously described (stored at 4 °C until processing)[17]. See Supplemental Methods for a detailed description of blood sample processing, and extraction of cfDNA and germline DNA.

### NGS library preparation

Deep targeted sequencing using a PC-tailored gene panel was employed to characterize alterations in cfDNA and germline DNA as previously described [13]. An overview of the panel is provided in Supplementary Table 1. Libraries were prepared using the Kapa Hyper Library Preparation Kit (KAPA Biosystems) and sequenced on an Illumina^Ⓡ^ Novaseq instrument (S-prime flowcell). Additional details are provided in Supplemental Methods. cfDNA libraries were also profiled by lpWGS, and ichorCNA was used to estimate ctDNA% [16, 17].

### Sequence alignment, initial processing, and quality control

Fastq files were demultiplexed and quality checked (fastQC, v. 0.11.8). Adapter sequences were trimmed (Skewer tool, v0.1.117) [18]. Paired-end sequences were mapped to the hg19 reference genome (BWA MEM, v.0.7.7)[19]. PCR and optical duplicates were removed (Picard markdups, v. 2.19)[20] followed by realignment (GATK4, 4.1.2.0) [21], structural variant calling, copy number analysis (additional details below), and MSI analysis. Ploidy and tumor purity were assessed using PureCN (v. 1.2.3) [22].

### Somatic variant calling and interpretation

SNVs and small insertions and deletions (indels) were called using 4 different tools: GATK Mutect2 (v. 4.1.2.0) [23], Strelka2 Somatic (v. 2.9.10) [24], VarDict (v. 1.6) [25], and VarScan2 (v. 2.4.2) [26]. Patient-matched germline samples were used for filtering. Additional filtration details are provided in the Supplemental Methods. Evidence of loss of heterozygosity (LOH) was assessed based on cfDNA copy number profiles and allele ratio of heterozygous single nucleotide polymorphisms (SNPs). All variants were manually inspected in the Integrative Genomics Viewer (IGV, v. 2.5.3).

Copy number variations (CNVs) were called using CNV Kit (v. 0.7.9) [27] and PureCN (v. 1.2.3) [22]. Somatic focal amplifications were called if the median log_2_-ratio at a given gene exceeded control regions (defined as 3–8 Mb up- and downstream of gene start/end, respectively) by ≥ 0.5. Likewise, somatic focal deletions were called when the log_2_-ratio of control regions exceeded that of the gene by ≥ 0.3. All somatic amplifications and deletions underwent manual curation in IGV (v. 2.5.3) and were considered real if supported by the SNP allele ratio. Homozygous deletions (log_2_-ratio ≤ −1) were defined as previously described [13].

Structural variants (SVs) were called using Svcaller (v. 1.0), SviCT (v. 1.0.1) [28], LUMPY (v. 0.3.0) [28], and SvABA (v. 1.1.0) [29]. Variants called by only one caller were discarded, except when only called by Svcaller. All variants were manually inspected in IGV.

Impact of SNVs and indels was annotated using Ensembl Variant Effect Predictor (ensemble-vep v. 96.0) [30]. Splice site alterations were further assessed for impact using multiple in silico tools (MaxEntScan, NNSplice) [31, 32]. SVs and CNVs were not evaluated for impact. Variants were annotated as pathogenic or likely pathogenic based on the databases ClinVar or OncoKB [33, 34] or introduction of a premature stop or frameshift in the coding sequence.

mSINGS (v. 3.6) [35] was used for MSI analysis. Samples with a mSINGS fraction ≥ 0.2 were annotated as having MSI.

To estimate the fraction of cfDNA that is tumor derived (ctDNA%), tumor cell purity was calculated using somatic SNVs with moderate or high impact. Additional details provided in Supplemental Methods. IchorCNA was additionally used to estimate ctDNA% [16, 17].

### Germline variant calling and interpretation

GATK Haplotypecaller (v. 4.1.2.0) [21] and Strelka2 Germline (v. 2.9.10) [24] were used to call germline SNVs and indels. Only germline variants with variant allele frequency (VAF) > 0.4, moderate or high impact, and allele frequencies of < 0.005 in gnomAD were considered. CNVs were assessed using CNV Kit (v. 0.7.9) [27] and PureCN (v.1.2.3) [22]. Deletions were defined as segmented log_2_-ratios of −1. All variants were manually inspected in IGV and annotated following the American College of Medical Genetics and Genomics (ACMG) guidelines [36].

### Clonal hematopoiesis

Likely clonal hematopoiesis (CH) variants were called based on the targeted sequencing of DNA from the buffy coat using GATK Haplotypecaller and Strelka2 Germline [21, 24], and annotated with Ensembl Variant Effect Predictor [30]. CH variants were filtered out from both baseline and progression samples. Additional details provided in Supplemental Methods and Supplementary Fig. 5.

### ddPCR analyses

Droplet digital PCR (ddPCR) assays targeting two resistance-associated SNVs in *AR* (Thr878Ala and Leu702His) were designed to assess the longitudinal dynamics of these mutations in plasma during first-line treatment. Additionally, for three mutations detected in matched baseline and progression samples by targeted sequencing (*TP53*_Arg209LysTer6, *PIK3CA*_Glu542Lys, *PIK3CA*_Glu545Lys), we used ddPCR assays previously designed *in-house* [37]. Additional details provided in Supplemental Methods and Supplementary Table 6.

### Clinical outcomes and statistical analysis

See Supplemental Methods for detailed description. Briefly, the primary endpoint was PSA PFS, defined as the time from first-line treatment initiation until time of PSA progression. PSA progression was confirmed as per PCWG3 criteria, by a second PSA measurement, 3 or more weeks later. PSA progression was also confirmed by imaging (radiographic progression) in all patients. As a secondary endpoint, we used OS defined as the time from treatment initiation till death from any cause. For clinical outcome analyses only pathogenic and likely pathogenic SNVs, SNVs annotated as high impact variants [21], amplifications, and homozygous deletions were included (i.e., structural variants and heterozygous deletions were excluded). Statistical analyses were conducted in R (v. 3.6.3) and in GraphPad Prism 9 (v. 9.5.1) with two-sided p-values < 0.05 considered as statistically significant.

### Clinically actionable alterations

Clinical actionability was annotated for all variants according to the knowledge base OncoKB, as previously described [34, 38, 39], considering actionability regarding targeted therapy, in a PC-specific manner. OncoKB classifies actionable alterations into different levels, based on the extent of evidence available for each as a marker of response to treatment (see Fig. 5A for description of levels). Level 4 alterations were considered not clinically actionable. These alterations are ones for which there is compelling biological evidence that the biomarker is predictive of response to a drug, however generally based solely on preclinical data. Similarly all variants were also annotated employing the ESMO Scale for Clinical Actionability of molecular Targets (ESCAT) [40, 41], which provides an evaluation of actionability in a European context.

## Results

### mCRPC cohort

Patients were enrolled at the time of mCRPC diagnosis at two tertiary hospitals in Denmark, and were further selected for this study based on having a ctDNA% of 3 or more, as estimated by ichorCNA [16]. We and others, have previously shown that higher ctDNA fraction is associated with worse outcomes in patients with mCRPC [17, 42, 43], and as such the current cohort represents patients with more aggressive disease (Supplementary Fig. 1). Patient characteristics are summarized in Table 1 and Supplementary Table 2. Approximately 50% of the patients had metastastic disease at the time of prostate cancer diagnosis. None of the patients had received prior treatment with androgen receptor pathway inhibitors. The majority (71.7%) had received solely androgen deprivation therapy prior to inclusion, and 18.9% had received upfront docetaxel treatment. A total of 71.7% (38/53) of patients received enzalutamide as first-line treatment, whereas the remaining patients were treated with abiraterone. At the time of last follow-up, 88.7% (47/53) of patients had experienced PSA progression on first-line treatment and 69.8% (37/53) had died.

**Fig. 1:**
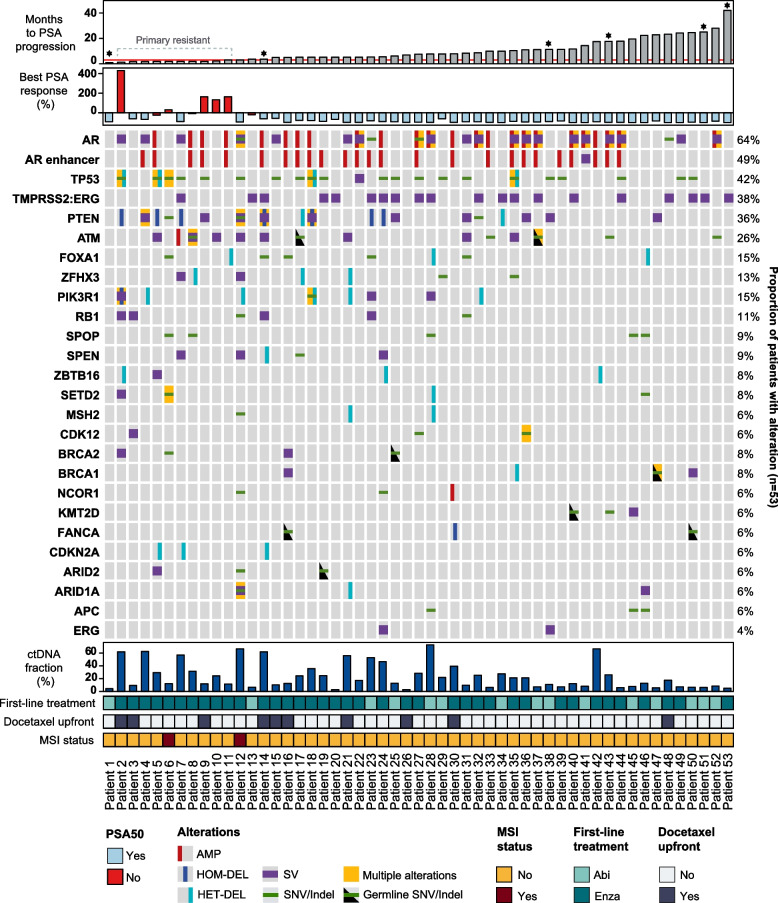
Overview of the most frequently altered genes in baseline samples from mCRPC patients. Genes with somatic or germline alterations in at least 3 patients are displayed. Only pathogenic and likely pathogenic SNVs, SNVs annotated as moderate or high impact, structural variants, amplifications, and heterozygous and homozygous deletions are shown. Patients are ordered according to months to PSA progression (top barplot, red line indicates cut-off for primary resistance defined as treatment failure within the first three months). ctDNA fraction as determined by ichorCNA is shown in the bottom barplot. *Indicates patients that were censored (see also Table 1). (AMP, amplification; HET-DEL, heterozygous deletion; HOM-DEL, homozygous deletion; MSI, microsatellite instability; SNV, small nucleotide variant; SV, structural variant)

A total of 19.2% (10/52) of patients exhibited primary resistance to first-line treatment, defined as treatment failure by 3 months (Fig. 1). One patient was excluded as he switched treatment due to side-effects (Patient 1). 30% of patients (3/10) categorized as having primary resistance achieved PSA50, within the first month, but rapidly progressed by 3 months (Fig. 1). None of the ten patients achieved PSA90. In contrast, 97.6% (41/42) of the patients not categorized as having treatment resistance achieved PSA50, and 64.3% (27/42) achieved PSA90.

### Deep targeted sequencing of PC-associated genes in plasma ctDNA and buffy coat DNA

Targeted sequencing was successfully performed on all samples resulting in median coverages of 1006X (range: 310-1987X) for plasma samples and 306X (range: 253-728X) for buffy coat samples. Somatic and germline SNVs and indels, structural variants and copy number alterations were called and curated manually in IGV as described in the Methods section. SNVs and indels were annotated for impact using Ensembl Variant Effect Predictor. Pathogenicity was annotated based on the databases ClinVar or OncoKB [33, 34] or introduction of a premature stop or frameshift in the coding sequence. Only pathogenic and likely pathogenic variants, and variants predicted to be of high or moderate impact, were further considered (detailed further in Methods and Supplemental Methods sections). Likely CH variants detected in the buffy coat were filtered away from ctDNA analyses, both in baseline and progression samples.

Median ctDNA% was 12.9% (range: 3.0–72.8%) at baseline and 16.1% (range: 3.0–64.8%) at progression based on ichorCNA analysis of matched lpWGS [16, 17]. These estimates were highly positively correlated to ctDNA% estimates from the deep targeted sequencing data, obtained from the VAF of likely driver mutation(s), defined as the somatic mutation(s) with the highest VAF and with moderate/high impact on protein function (rho = 0.849, p < 0.0001, Spearman’s rank correlation test). For simplicity, we present only the ctDNA% estimates based on ichorCNA analyses throughout the manuscript.

All but one patient (98.0%, 52/53) had at least one somatic or germline variant identified at baseline, and all had at least one at progression (100%, 18/18). The patient without any variants at baseline had a ctDNA% of 4.5% (Patient 1) (Fig. 1). The most frequently altered gene at baseline was *AR,* with amplification detected in 45.3% (24/53) of patients and SVs in 35.8% (19/53) of patients (Fig. 1). The *AR* enhancer region was also commonly amplified (25/53, 47.2%), and this often co-occurred with amplification of the *AR* gene (20/25, 80.0%). In total, 67.9% (36/53) of patients had at least one genomic alteration in the *AR* region at mCRPC diagnosis.

Other commonly detected alterations at baseline included somatic SNV/indels, copy number losses and SVs in *TP53* (22/53, 41.5%), *TMPRSS2:ERG* fusions (20/53, 37.7%), and somatic SNV/indels, copy number losses and SVs in *PTEN* (19/53, 35.8%), as well as a broad range of both somatic and germline alterations in *ATM* (14/53, 26.4%) (Fig. 1). Of note, 50.9% (27/53) of patients had at least one somatic or germline alteration in the homologous recombination repair (HRR) pathway, defined as genes included in the PROfound trial [44], i.e., *BRCA1, BRCA2, ATM, BRIP1, BARD1, CDK12, CHEK1, CHEK2, FANCL, PALB2, PPP2R2A, RAD51B, RAD51C, RAD51D,* and *RAD54L,* as well as *NBN, RAD50, FANCA, ATR* and *MRE11*.

Furthermore, 3.8% (2/53) of patients were found to have microsatellite instability (MSI) (Fig. 1). One of the two patients with MSI had a homozygous deletion of *MLH1*, as well as a missense variant of unknown significance in *MSH2* (Gln252Arg), and the other had a missense variant of unknown significance in *MSH3* (Thr230Ala).

Germline alterations were detected in 22.6% (12/53) of patients, with two of these harboring alterations in two genes (Supplementary Fig. 2). Of these, only 6 patients (11.3%, 6/53) had pathogenic or likely pathogenic variants according to ACMG classification; most commonly in *ATM* (2/53, 3.8%). At total of 15.1% (8/53) of patients had germline alterations in HRR genes, of which 5 patients had pathogenic or likely pathogenic variants (9.4%, 5/53). The remainder were variants of uncertain significance, however predicted to have high/moderate impact on function. All germline alterations detected were SNVs.

Variants likely to be CH were detected in the buffy coat samples of 7 patients (7/53, 13.2%) at either baseline and/or progression (Supplementary Table 3). Variants were noted in *BRCA2*, *CHEK2*, *DNMT3A*, *KMT2C*, *TP53* and *SF3B1*. The variants in *TP53* and *SF3B1* were classified as clonal hematopoiesis of indeterminate potential (CHIP) variants, defined as pathogenic variants with a VAF ≥ 2% in individuals without evidence of hematologic malignancy, dysplasia, or cytopenia [45]. All likely CH/CHIP variants could be found in the matching plasma circulating cell-free DNA sample as well, with generally comparable VAFs (Supplementary Table 3).

### Genomic correlates of clinical outcomes

To identify candidate prognostic biomarkers we first investigated for potential associations between the most commonly identified alterations in our cohort at baseline (amplification of *AR/AR* enhancer, *PTEN* and *TP53* alterations) and patient outcomes defined by PSA progression free survival (PFS) and overall survival (OS).

**Fig. 2:**
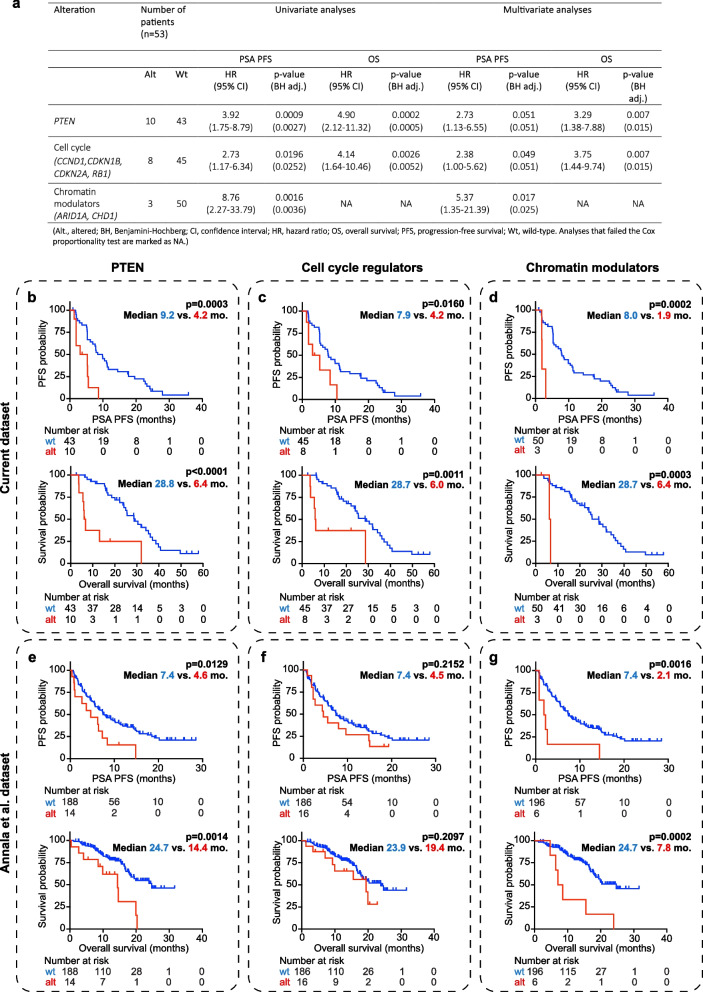
Genomic correlates of clinical outcomes. **a **Univariate and multivariate Cox regression using PSA PFS and OS as endpoints. Multivariate analyses corrected for ctDNA fraction. **b**-**d **Kaplan Meier plots of patients with alterations in *PTEN*, cell cycle regulators (*CCND1, CDKN1B, CDKN2A, RB1*), or chromatin modulators (*ARID1A, CHD1*), compared to patients without alterations, using PSA PFS and OS as endpoints. **e**-**g **Kaplan-Meier plots for same genes as in b-d, however employing publicly available data from Annala et al. [48]. *P*-values in Kaplan-Meier plots based on log-rank test. Only pathogenic and likely pathogenic SNVs, SNVs annotated as high impact variants, amplifications, and homozygous deletions were included (i.e., structural variants and heterozygous deletions were excluded)

*AR/AR* enhancer amplification and *TP53* alterations were not statistically significantly associated with PFS or OS in our cohort (BH adj. *p* > 0.05, univariate and multivariate cox regression analysis, Supplementary Table 4). In contrast, alterations in *PTEN* were significantly associated with shorter PFS in univariate cox regression analysis (HR = 3.92, 95% CI: 1.75 − 8.79, BH adj. *p* = 0.0027, Fig. 2A) and were borderline significant after adjustment for ctDNA% (HR = 2.73, 95% CI: 1.31–6.55, BH adj. *p* = 0.051, Fig. 2A). Alterations in *PTEN* were also associated with significantly worse OS in univariate cox regression analysis (HR = 4.90, 95% CI: 2.12–11.32, BH adj. *p* = 0.0005, Fig. 2A), also after adjusting for ctDNA% (HR = 3.29, 95% CI: 1.38–7.88, BH adj. *p* = 0.015, Fig. 2A). In agreement with this, Kaplan Meier analyses showed that median PFS was significantly shorter for patients with alterations in *PTEN* (4.2 months vs. 9.2 months, log-rank test, *p* = 0.0003, Fig. 2B), as was median OS (6.4 months vs. 28.8 months, log-rank test, *p* < 0.0001, Fig. 2B).

We further examined the biomarker potential of several pathways commonly altered in mCRPC, including AR signaling, cell cycle regulation, WNT signaling, the PI3K pathway, the HRR pathway, and chromatin modulation by grouping genes into gene sets [46–48]. Alterations in genes associated with cell cycle regulation (*CCND1*, *CDKN1B*, *CDKN2A*, and *RB1*) were associated with significantly shorter PFS (univariate Cox regression, HR = 2.73, 95% CI: 1.17 − 6.34, BH adj. *p* = 0.0252, Fig. 2A), and were borderline significantly associated with worse PFS after correcting for ctDNA% (HR = 2.38, 95% CI: 1.00–5.62, BH adj. *p* = 0.051, Fig. 2A). Alterations in genes involved in cell cycle regulation were also associated with significantly shorter OS (univariate Cox regression analysis, HR = 4.14, 95% CI: 1.64–10.46, BH adj. *p* = 0.0052, Fig. 2A), which remained significant after adjusting for ctDNA% (HR = 3.75, 95% CI: 1.44–9.74, BH adj. p = 0.015, Fig. 2A). Kaplan Meier analyses furthermore showed that median PFS was significantly shorter for patients with alterations in cell cycle regulators (4.2 vs. 7.9 months, log-rank test, *p* = 0.0160, Fig. 2C), as was median OS (6.0 months vs. 28.7 months, log-rank test, *p* = 0.0011, Fig. 2C).

Alterations in chromatin modulators (*ARID1A*, *CHD1*) were additionally found to be significantly associated with poorer PFS in univariate Cox regression (HR = 8.76, 95% CI: 2.27- 33.79, BH adj. p = 0.0036, Fig. 2A), also after adjusting for ctDNA% (HR = 5.37, 95% CI: 1.35–21.39, BH adj. *p* = 0.025, Fig. 2A). The chromatin modulator gene set failed the Cox proportionality test with OS as an endpoint. Median PFS was also significantly shorter for patients with alterations in chromatin modulators (1.9 months vs. 8.0 months, log-rank test, p = 0.0002, Fig. 2D), as was median OS (6.4 months vs. 28.7 months, log-rank test, *p* = 0.0003, Fig. 2D).

Alterations in the PI3K signaling pathway were associated with poorer PFS and OS in univariate Cox regression, although this was no longer statistically significant upon Benjamini–Hochberg correction, nor was it significant when adjusting for ctDNA% (Supplementary Table 4). Of note this association was mainly driven by alterations in *PTEN* (Fig. 2, Supplementary Table 4). Alterations in *AR* signaling, the HRR pathway and WNT signaling were not associated with PFS or OS in univariate or multivariate Cox regression (BH adj. *p* > 0.05, Supplementary Table 4).

In summary, in our mCRPC cohort, we found significant associations between poor PFS and poor OS and genomic alterations in *PTEN*, cell cycle regulator genes, and chromatin modulator genes (Fig. 2A-D). For independent clinical validation, we employed publicly available data from Annala et al. [48], who performed genomic profiling of ctDNA from 202 mCRPC patients prior to starting enzalutamide or abiraterone treatment. We successfully validated the association between alterations in *PTEN* and chromatin modulators and worse PFS in this independent cohort (log-rank test, *p* = 0.0129 and *p* = 0.0016, respectively), as well as worse OS (log-rank test, *p* = 0.0014 and p = 0.0002, respectively) (Fig. 2E, G). These alterations were also associated with worse outcomes (PFS, OS) in univariate Cox regression (Supplementary Table 5), however not after adjusting for ctDNA %. Shorter PFS and OS were noted for patients with alterations in cell cycle regulators in the Annala et al. cohort as well, although not statistically significant (log-rank test, p = 0.2152 for PFS; log-rank test, p = 0.2097 for OS, Fig. 2F, and univariate Cox regression in Supplementary Table 5).

### Genomic candidate biomarkers of intrinsic resistance to second-generation anti-androgens

We further explored whether certain alterations detected in our mCRPC cohort were enriched for in patients with primary resistance to enzalutamide or abiraterone acetate treatment (non-responders), compared to patients sensitive to treatment (responders). Primary resistance was defined as treatment failure by 3 months. For independent validation, we again used the dataset from Annala et al. [48].

**Fig. 3:**
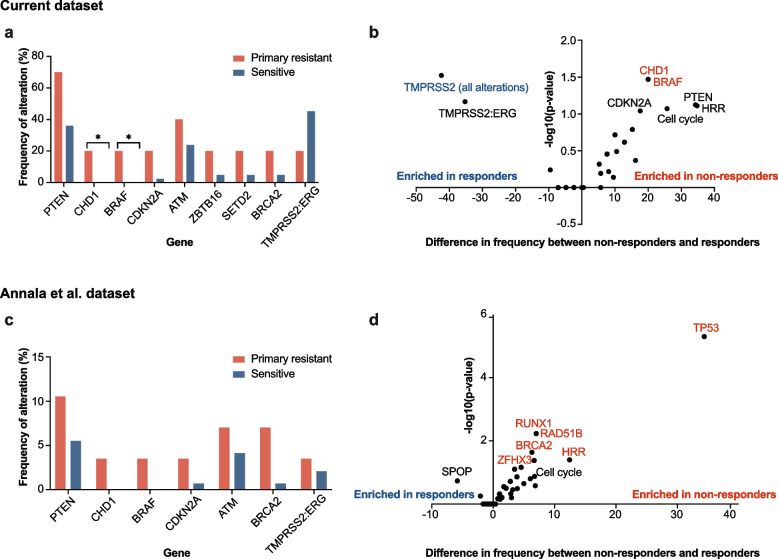
Genomic correlates of intrinsic resistance to second-generation anti-androgens. **a **Frequency of alterations in patients with primary resistance to first-line enzalutamide or abiraterone acetate compared to patients without. Primary resistance was defined as treatment failure by 3 months, and only genes with alterations in at least two patients were considered. (Fisher’s exact test, *p-value < 0.05) **b** Comparison of the mutational frequencies of alterations in patients with primary resistance to enzalutamide or abiraterone acetate relative to patients that responded to treatment. The difference in relative frequency is shown on the x-axis and the -log10(*p*-value) (Fisher’s exact test) is shown on the y-axis. Genes that were significantly enriched for in non-responders are shown in red and those enriched for in responders in blue (*p*-value < 0.05). **c** Frequency of alterations in patients with primary resistance for genes in (a) based on the Annala et al. cohort [48] **d** Analysis as in (b), however based on the Annala et al. dataset

In our cohort, *BRAF* amplification and loss of *CHD1* (a chromatin modulator) were only found in patients with primary resistance (20.0% of non-responders compared to 0% of responders for both genes, *p* < 0.05, Fisher’s exact test, Fig. 3A; enriched in non-responders (*p* < 0.05, Fig. 3B). This was also the case in the Annala et al. dataset, with *BRAF* alterations and *CHD1* loss only being identified in patients with primary resistance to first-line treatment with enzalutamide or abiraterone, although this did not reach statistical significance (both *p* = 0.08, Fisher’s exact test, Fig. 3C). Furthermore, alterations in HRR genes were enriched for in non-responders in both our dataset and the Annala et al. dataset, but only statistically significant in the latter (Fig. 3B and Fig. 3D). *TP53* alterations were markedly enriched for in the Annala et al. dataset in non-responders (54% vs. 20% in responders, *p* = 5 × 10^–6^), but not in our dataset (50% with alterations in non-responders vs. 40% in responders, *p* = 0.73). This divergence may be attributed to a variety of factors, such as our relatively limited population size, differences in the composition of the patient cohorts (e.g. 43% of patients in Annala et al. had undetectable ctDNA levels) or differences in the assays employed for ctDNA sequencing.

Interestingly, we found that *TMPRSS2* fusions, primarily composed of *TMPRSS2:ERG* fusions, were strongly enriched for in responders (Fig. 3B, *p* < 0.05, Fisher’s exact test). There were only few patients with *TMPRSS2* or *ERG* alterations in the Annala et al. dataset.

Lastly, we noted that one patient in our cohort with primary resistance had a pathogenic variant in *CTNNB1* likely leading to WNT pathway activation, which has previously been associated with resistance to second-generation anti-androgens [6] (data not shown, Patient 5). Additionally, we noted biallelic *TP53* and *RB1* loss in one patient with primary resistance, which has been associated with a neuroendocrine phenotype and resistance to second-generation anti-androgens [49] (Fig. 1, Patient 2).

### Genomic changes in AR during first-line treatment with second generation anti-androgens

**Fig. 4:**
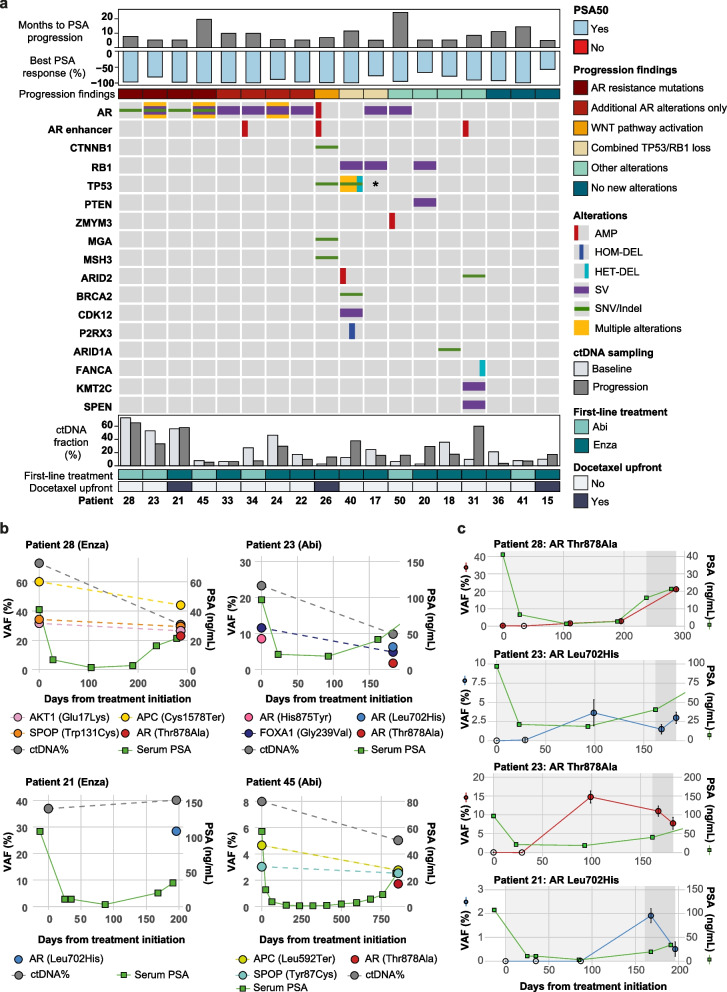
Emerging genomic alterations during first-line treatment with second generation anti-androgens. **a **Oncoplot of variants emerging at progression (*n *= 18). **b **Overview of variants in matched baseline and progression samples from four patients that acquired *AR* resistance mutations at progression. VAFs for the corresponding variants, as well as changes in PSA and ctDNA% from baseline to progression are shown. **c **Patient-specific ddPCR assays showing the longitudinal dynamics of *AR* resistance mutations. VAF based on ddPCR is shown, as well as PSA changes from baseline to progression. Open circles represent time points where the variant was not detected based on ddPCR. Shaded region indicates time from initial PSA progression to treatment discontinuation. (AMP, amplification; HET-DEL, heterozygous deletion; HOM-DEL, homozygous deletion; SNV, small nucleotide variant; SV, structural variant). *SNV present at baseline

To identify genomic alterations that may be associated with acquired resistance to enzalutamide or abiraterone acetate, we performed targeted sequencing of plasma samples drawn at progression (at time of treatment cessation) for 18 of the 53 patients (Fig. 4, Supplementary Fig. 3). Of note, three of the 18 patients had no additional genomic alterations identified at progression (Fig. 4A).

An increase in the proportion of patients with at least one alteration in the *AR* region was noted at progression compared to baseline (16/18 (89%) vs. 13/18 (72%), respectively, *p* = 0.40, Fisher’s exact test; Supplementary Fig. 3). The emergence of known resistance mutations [5] in *AR* (Thr878Ala (*n* = 3) or Leu702His (*n* = 2)) was noted in 22% of patients (4/18), with one patient acquiring both mutations (Figs. 4A and B, Patient 23). Interestingly, *AR* resistance mutations appeared to be mutually exclusive with alterations in other genes likely conferring resistance (e.g. *CTNNB1* mutation, *TP53/RB1* loss Fig. 4A). Four additional patients had alterations solely in the *AR* region arising at progression, all four of which acquired structural variants in *AR* (Fig. 4A).

We went on to examine the longitudinal dynamics of these *AR* mutations employing mutation-specific ddPCR assays (Fig. 4C). We found that these alterations were in fact detectable in 2 of the 3 patients prior to detectable PSA increase (63–126 days earlier, Patient 28 and 23). In the remaining patient the mutation was detectable at the time of PSA increase (Patient 21). Of note, in Patient 28, the *AR* Thr878Ala mutation was also detectable by ddPCR at a very low VAF at the time of treatment initiation, suggesting the early presence of enzalutamide-resistant tumor cells. The patient had only been treated with LHRH (luteinizing hormone-releasing hormone) agonist previously. Higher sequencing depth would be required to call such low-VAF mutations using sequencing-based approaches.

### Additional emerging genomic alterations during first-line treatment

Besides mutations in *AR*, we detected emerging alterations in multiple other genes (Fig. 4A, Supplementary Fig. 3), including the activating Ser45Phe mutation in *CTNNB1,* which has previously been implicated in enzalutamide resistance [50, 51]. We furthermore detected the emergence of an SV of unknown impact in *RB1*, along with a heterozygous deletion of *TP53* and a splice variant of unknown significance in *TP53* (Patient 40, Fig. 4A), as well as the emergence of a structural variant in *RB1* in a patient with a *TP53* frameshift mutation at baseline (Patient 17). Combined loss of *RB1* and *TP53* has, as already mentioned, been reported to promote lineage plasticity to a neuroendocrine phenotype and resistance to antiandrogen therapy [49, 52]. Additional alterations identified have not previously been associated with resistance to second-generation androgens and may reflect novel resistance mechanisms (i.e., *ARID2*, *ARID1A*, *KMT2C*) (Fig. 4A, Supplementary Fig. 3).

As for *AR* mutations, we considered for a subset of patients, whether we could employ changes in SNVs detected at baseline, employing patient-specific ddPCR assays, to monitor treatment response (Supplementary Fig. 4). Overall, we found that changes in VAFs paralleled PSA dynamics, with SNV clearance potentially indicating greater response to treatment (Supplementary Fig. 4C and Supplementary Fig. 4D). At a single time point (Supplementary Fig. 4B, Patient 15), we observed decreases in the VAF despite increasing PSA values, the physiological and clinical significance of which is not clear. These results are preliminary, however highlight potential challenges in employing VAFs of single SNVs for monitoring response.

### Clinically actionable alterations in ctDNA of newly diagnosed mCRPC patients

**Fig. 5:**
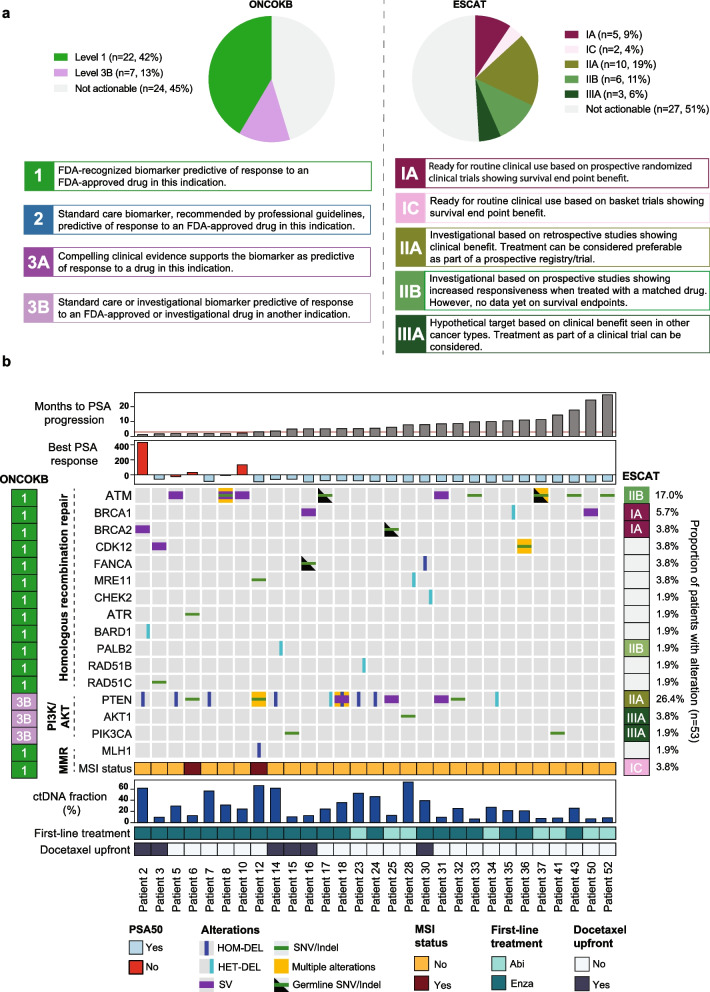
Clinically actionable alterations. **a **Proportion of clinically actionable alterations based on the highest-level alterations identified for each patient, annotated based on OncoKB and ESCAT (the different levels of clinically actionable alterations according to OncoKB are shown adapted from https://www.oncokb.org/, and for ESCAT from Mateo et al. [40] and Mosele et al. [41]. Only relevant tiers are noted herein). **b **Overview of clinically actionable alterations detected in the cohort and as annotated by OncoKB and ESCAT. (AMP, amplification; HET-DEL, heterozygous deletion; HOM-DEL, homozygous deletion; MMR, mismatch repair; SNV, small nucleotide variant; SV, structural variant)

OncoKB [34], a curated precision oncology knowledge database developed by Memorial Sloan Kettering Cancer Center (New York, USA), was used to classify variants into tiers of clinical actionability, as relating to treatment, in a PC-specific manner. A total of 54.7% (29/53) of patients had at least one clinically actionable alteration at baseline, including 41.5% with a level 1 alteration as the highest level alteration, and 13.2% with a level 3B alteration as the highest level (Fig. 5A). In addition, we classified variants with the ESMO Scale for Clinical Actionability of molecular Targets (ESCAT) [40, 41], to consider clinical actionability in a European setting as well, as OncoKB is based on an FDA-regulatory context. Employing ESCAT, 49.0% of patients had at least one clinically actionable alteration at baseline, however only 13.2% had tier 1 alterations. Differences were largely accounted for by alterations in the HRR pathway, that are FDA-recognized biomarkers predictive of response to PARP inhibition (as monotherapy or in combination with enzalutamide), but which have not received EMA approval (e.g. *ATM, CDK12, PALB2*).

The majority of clinically actionable alterations were in HRR genes (Fig. 5B). Additional clinically actionable alterations were identified in the PI3K/AKT pathway, and two patients had MSI phenotype. Thirteen patients (13/53, 24.5%) had clinically actionable alterations in multiple genes. Only four patients had clinically actionable alterations of germline origin (4/53, 7.5%), all of which were in HRR genes (Fig. 5B). Only few potential additional clinically actionable alterations were identified at the time of progression (one patient with heterozygous deletion of *FANCA*, and one with a structural variant in *PTEN* expected to result in a loss of function alteration) (Supplementary Fig. 3).

## Discussion

In this study we successfully sequenced plasma ctDNA and germline DNA (buffy coat) samples from a Danish cohort of 53 patients with mCRPC prior to commencing first-line treatment with enzalutamide or abiraterone acetate, and for a subset at progression, using deep targeted sequencing with a PC-tailored gene panel. In doing so we identified both known and novel predictive and prognostic biomarkers, as well as clinically actionable alterations providing additional therapeutic options. Our results provide real-world evidence of the significant potential of genomic profiling of ctDNA to inform treatment in patients with mCRPC.

Consistent with prior studies, loss of function alterations in *PTEN* and in cell cycle regulators (*CCND1*, *CDKN1B*, *CDKN2A*, *RB1*) were found to confer worse outcomes (PFS/OS) [47, 48, 53, 54]. We further found perturbations in chromatin modulators (*ARID1A*, *CHD1)* to be significantly associated with worse outcomes, with a median PFS of only 1.9 months compared to 8 months and a median OS of 6.4 months compared to 28.7 months, for patients with alterations vs. those without. These findings were validated in an external publicly available data set from Annala et al. [48]. Loss of *CHD1* has previously been linked to an increased risk of postoperative metastasis following radical prostatectomy and has been found to promote spontaneous metastasis formation in animal models of prostate cancer [55], suggesting that these alterations may be associated with more aggressive disease. *ARID1A* has not, to our knowledge, been reported to be associated with poor outcomes in patients with mCRPC. These findings warrant validation in larger cohorts.

A significant proportion of patients (19.2%) exhibited primary resistance to first-line enzalutamide or abiraterone acetate treatment. In these patients we detected enrichment for *BRAF* amplification and *CHD1* loss, as well as alterations well-known to confer resistance to second-generation anti-androgens (e.g. activating alterations in *CTNNB1,* and combined loss of *TP53* and *RB1)*. *BRAF* alterations and *CHD1* loss were also identified solely in patients with primary resistance in the Annala et al. dataset [48]. To our knowledge *BRAF* amplification has not previously been described in patients as a mechanism of resistance to enzalutamide or abiraterone treatment, however *BRAF* has been identified as a strong modulator of enzalutamide sensitivity in a CRISPR-Cas9 resistance screen, and activating *BRAF* mutations have been detected in patients with primary enzalutamide resistance [56]. *CHD1* loss has previously been found to be enriched in mCRPC patients with primary resistance to abiraterone and enzalutamide based on analysis of circulating tumor cells [57], and low CHD1 expression has been reported to be associated with shorter response to enzalutamide but not abiraterone [58]. Both patients with *CHD1* loss in our cohort were treated with enzalutamide. Together these data lend clinical validation to recent findings from an in vivo shRNA screen identifying *CHD1* loss as a key mediator of enzalutamide resistance [58]. Activating alterations in *CTNNB1* were detected, both in patients with primary resistance and patients progressing on treatment, consistent with prior clinical reports of WNT pathway activation promoting resistance to second-generation anti-androgens [4, 6]. Similarly, combined *TP53* and *RB1* loss was detected in one patient with primary resistance and one with a short-lived response to enzalutamide, as well as in one patient at the time of progression, consistent with multiple prior studies demonstrating that combined *TP53/RB1* loss promotes a shift to a neuroendocrine phenotype and resistance to antiandrogen therapy [49, 52]. Lastly, we noted enrichment for *TMPRSS2* (primarily *TMPRSS2:ERG*) alterations in patients that responded to enzalutamide or abiraterone treatment. This is consistent with data from an in vivo bone tumor growth model showing that enzalutamide treatment is more effective in tumours expressing ERG [59], as well as with an earlier clinical study demonstrating that patients having PSA decline during abiraterone treatment were significantly more likely to have ERG rearrangements [60]. It is further in line with recently published data from the ProBio trial demonstrating that patients with *TMPRSS2:ERG* fusions benefit longer from androgen receptor pathway inhibitor treatment than those without these alterations [15]. Taken together these results strongly suggest that genomic profiling of ctDNA prior to commencing first-line treatment with second-generation anti-androgens may be of value in identifying patients that are unlikely (or more likely) to respond to treatment.

Additional alterations that emerged during first-line treatment included mutations in the *AR* gene that are known to be involved in resistance to second-generation anti-androgens (e.g. *AR* Thr878Ala). These mutations were found to be mutually exclusive with other alterations believed to confer resistance, such as *CTNNB1* activating mutations and combined *TP53/RB1* loss. Other alterations identified at progression have not previously been linked to resistance to enzalutamide or abiraterone (e.g. *ARID2*, *ARID1A*, *KMT2C* loss), and further studies are needed to determine whether these play a role in resistance acquisition or whether these are passenger mutations. Three of the 18 patients had no additional genomic alterations identified at progression, suggesting additional resistance mechanisms not detected with our targeted gene panel. These results emphasize significant heterogeneity in developing resistance to treatment with second-generation anti-androgens and highlight the potential of genomic profiling of ctDNA in detecting mechanisms of acquired resistance in the setting of first-line treatment of mCRPC. Our results furthermore provide clinical validation of resistance candidates (e.g. *BRAF, CHD1*) identified in prior in vitro and in vivo resistance screens with second-generation anti-androgens.

Employing ddPCR we demonstrated that AR resistance mutations could be detected several months prior to PSA increases, including in one patient prior to initiation of an androgen receptor pathway inhibitor. These results are preliminary and the clinical consequences are unclear (i.e. optimal timing for switching treatment), however these results warrant further investigation. In contrast, we found that monitoring treatment response, by following the VAFs of single SNVs detected at baseline, largely paralleled PSA dynamics, suggesting that this approach does not provide added clinical utility.

About half of the patients in our cohort had a clinically actionable alteration, in particular in HRR genes, and thus could potentially be eligible for treatment with a PARP inhibitor (monotherapy or in combination with a second-generation anti-androgen). It is however important to note that the degree of response to PARP inhibition varies across the HRR genes, with the greatest response seen in patients with *BRCA1/2* loss and less in those with for instance *ATM*, *CDK12* or *CHEK2* loss-of-function alterations [61–63]. Further studies are needed to optimize selection of patients and improve treatment strategies in patients with non-*BRCA* HRR alterations. Of further note, a significant proportion of patients in our cohort had loss-of-function alterations in *PTEN* (26%), which may benefit from combination therapy with a second-generation anti-androgen and an AKT inhibitor. A recent phase III trial demonstrated significantly improved radiographical PFS with combined abiraterone and ipatasertib (AKT inhibitor) treatment vs. abiraterone alone in mCRPC patients with *PTEN* loss [64], and possibly improved overall survival (genomic *PTEN* loss, HR: 0.76, 95% CI 0.54–1.07; and *PIK3CA*/*AKT1*/*PTEN* alterations, HR: 0.70, 95% CI 0.51–0.96) [65]. Clinical trials with additional AKT inhibitors are underway. The majority of clinically actionable alterations identified in our study were somatic, however 7.5% of patients had germline alterations, which bear additional implications with regards to genetic counselling of the patient and family members. This highlights the need for both somatic and germline testing in this population. Interestingly, few additional clinically actionable alterations were identified at the time of progression, which is in line with recent evidence suggesting that there is limited evolution of the actionable metastatic cancer genome under therapeutic pressure [66]. Lastly, our findings contrast the landscape of clinically actionable alterations in an American, as well as a European context, and identify considerably fewer level 1 alterations based on ESCAT in this dataset (41.5% vs. 13.2%, OncoKB vs. ESCAT). This difference was largely driven by HRR genes, as 42% of patients had a level I alteration in HRR genes based on OncoKB, while only 9% had a level I alteration based on ESCAT (restricted to *BRCA1/2* loss-of-function alterations). This suggests in part a more conservative evaluation of actionability and of regulatory drug approvals in Europe.

Our study further confirms that a significant proportion of patients with mCRPC have CH variants, as 9.4% of patients harbored such variants at baseline and 15.8% at progression [13, 67]. Although none of the specific CH variants identified in our cohort were classified as clinically actionable, some occured in clinically actionable genes (i.e., *BRCA2, CHEK2*), emphasizing the importance of including matched germline samples when performing deep sequencing of plasma cfDNA samples, to filter CH variants in order to avoid false positive somatic variants.

Lastly, our cohort only consists of 53 patients and only patients with more than 3% ctDNA at baseline. The cohort represents patients with more aggressive disease and shorter PFS/OS. The proportion of certain HRD alterations was higher in our cohort than in previously reported mCRPC populations [68, 69], e.g. *BRCA1* and *ATM*. We speculate that this may be attributed to having selected patients with ctDNA% of 3 or more, as we, and others, have previously shown that prostate cancer patients with germline DNA repair gene alterations have poorer outcomes (in particular *BRCA2* and *ATM* germline variants) [70, 71], and this may also be the case for somatic alterations [72, 73]. Targeted analysis of patients with low ctDNA% is warranted to ensure a more representative cohort. The cohort furthermore represents a relatively homogenous Danish population, recruited at only two Urology departments, and results may in part reflect clinical practice at these departments at the time of recruitment (2016–2018). Nevertheless, despite the homogenous population, the modest population size, and the exclusion of patients with low ctDNA levels, the genomic profile of our population is similar to that in other published studies profiling patients with mCRPC [68, 69, 74]. Additionally several of the prognostic and predictive markers identified have also been identified in prior studies of mCRPC populations [47, 48, 53, 54], as well as validated in the current study employing the cohort from Annala et al. [48]. A further limitation of the current study is that a panel of genes known to be of relevance in PC was employed, thus not permitting the discovery of entirely novel biomarkers.

## Conclusion

Genomic profiling is not routinely performed to guide treatment in patients with newly diagnosed mCRPC. Our understanding of the genomic landscape of prostate cancer has however significantly expanded in recent years, as has the development of targeted treatments requiring specific genomic alterations for eligibility. Our study demonstrates that deep targeted sequencing of plasma ctDNA has significant clinical potential to inform treatment in the first-line setting, including identifying patients that are unlikely to respond to treatment, providing prognostic information, detecting mechanisms of resistance emergence during treatment, and informing on additional targeted therapy options. Additional larger prospective studies are urgently needed to address the clinical utility, and optimized implementation, of these ctDNA-based biomarkers in the management of mCRPC.

## Supplementary Information

Supplementary Material 1.

## Data Availability

The lpWGS and targeted sequencing data generated in this study is available through controlled access from GenomeDK (https://genome.au.dk/). Accession number GDK000012. (https://genome.au.dk/library/ GDK000012/)).

## Abbreviations

ACMG: American College of Medical Genetics and Genomics
Alt: Altered
Amp: Amplification
ARSI: Androgen signaling inhibitors
BH: Benjamini-Hochberg
cfDNA: Circulating cell-free DNA
CH: Clonal hematopoiesis
CHIP: Clonal hematopoiesis of indeterminate potential
CNV: Copy number variant
ctDNA: Circulating tumor DNA
ddPCR: Droplet digital PCR
ESCAT: ESMO Scale for Clinical Actionability of molecular Targets
Hom-del: Homozygous deletion
LPWGS: Low-pass whole genome sequencing
MAF: Mutant allele frequency
mCRPC: Metastatic castration-resistant prostate cancer
MSI: Microsatellite instability
OS: Overall survival
PARP: Poly-ADP-Ribose-Polymerase
PBMC: Peripheral blood cell mononuclear cells
PC: Prostate cancer
PSA PFS: PSA progression-free survival
SNP: Single nucleotide polymorphisms
SNV: Single nucleotide variant
SV: Structural variant
UMI: Unique molecular identifier
VAF: Variant allele frequency
WT: Wild-type
