# Supplementary material for: Deep targeted sequencing of circulating tumor DNA to inform treatment in patients with metastatic castration-resistant prostate cancer
Source: J Exp Clin Cancer Res. 2025 Apr 14;44:120. doi: 10.1186/s13046-025-03356-0 (PMC11998381; doi:10.1186/s13046-025-03356-0)
Supplement: Supplementary file 1 — Supplementary Material 1. [file 13046_2025_3356_MOESM1_ESM.zip › Supplementary Materials/Supplemental Methods.pdf]

Supplemental Methods:

Blood sample processing and extraction of circulating free DNA (cfDNA) and germline DNA

Blood samples were collected in 10 mL BD Vacutainer K_2 EDTA tubes (Beckton Dickinson) and processed within 2 hours as previously described (stored at 4°C until processing) (1). To separate plasma from cellular components, blood samples were centrifuged at 3000*g* for 10 minutes (20°C). Plasma and buffy coat samples (peripheral blood mononuclear cells, PBMCs, used as germline reference) were stored in cryotubes (TPP) at -80°C.

cfDNA was extracted from 2.0-4.5 mL plasma on a QIAsymphony robot (Qiagen) using the QIAamp Circulating Nucleic Acids kit (Qiagen). Extracted cfDNA was stored in LoBind tubes (Eppendorf AG) at -80°C until further analysis (<1 month). cfDNA extraction efficiency was evaluated employing a CPP1 spike-in ddPCR assay, and potential contamination with DNA from lysed PBMCs was evaluated by a ddPCR assay targeting the VDJ rearranged immunoglobulin heavy chain locus, specific for B lymphocytes, as previously described (2). Median extraction efficiency was 68.6% (range: 46.0-86.7%). Contamination with PBMC genomic DNA (≥3 positive droplets on ddPCR) was not observed in any of the samples.

Germline DNA was extracted from buffy coats (400 µL) using the QiaSymphony DP DNA Mini Kit (Qiagen).

DNA concentrations were determined using Qubit fluorometric quantification (dsDNA Broad range, ThermoFisher).

NGS library preparation

Deep targeted sequencing using a PC-tailored gene panel was employed to characterize alterations in cfDNA and germline DNA as previously described (3). Briefly, germline DNA was sheared into shorter fragments (~250-350 bp) prior to library preparation, using a Covaris E220 Evolution ultrasonicator (80 seconds, Covaris). Fragment size was evaluated using the Tapestation 4200 system (HSD1000 Screentape assay, Agilent Technologies). Libraries were prepared for both cfDNA (11-50 ng) and germline DNA (50 ng) using the Kapa Hyper Library Preparation Kit (KAPA Biosystems) as per the manufacturer's guidelines, however all reagent

amounts were halved due to the low input of DNA. Each PCR reaction was split into four reactions to avoid PCR error overtake and improve the conversion of raw reads to unique reads. PCR reactions were pooled again after amplification. xGen CS-adapters – Tech Access (IDT-DNA) with unique molecular identifiers on both strands were used along with primers containing unique indexes on both strands. Indexed libraries were quantified using Qubit and fragment lengths were evaluated using the Tapestation 4200 (D1000 Screentape assay, Agilent Technologies). Libraries (cfDNA, germline) were pooled equimolarly (8-plex) for in-solution target enrichment using Twist Bioscience's Custom Target Enrichment followed by real-time PCR (CFX connect, Bio-Rad). The resulting pools were quantified using Qubit (dsDNA HS quantification, ThermoFisher) and fragment lengths were evaluated using BioAnalyzer (dsDNA HS Kit, Agilent Technologies). The resulting pools were then combined equimolarly and paired-end sequenced (2x100bp) on an Illumina Novaseq instrument (S1 flowcell).

Somatic variant calling and interpretation

Somatic single nucleotide variants (SNVs) and small insertions and deletions (indels) were called using 4 different tools: GATK Mutect2 (v. 4.1.2.0)(4), Strelka2 Somatic (v. 2.9.10)(5), VarDict (v. 1.6)(6), and VarScan2 (v. 2.4.2)(7). Patient-matched germline samples were used for filtering. For additional filtration of somatic SNVs, the following inclusion criteria were used: supported by ≥ 10 reads, called by ≥3 callers, and annotated as pathogenic or likely pathogenic in the databases ClinVar or OncoKB (8, 9) or introduction of a premature stop or frameshift in the coding sequence. Variants of high or moderate impact called by only 2 callers, and those with VAF (Variant allele frequency) <0.02 that were high or moderate impact and called by 4 callers or detected in another sample from the same individual, were also kept. Evidence of loss of heterozygosity (LOH) was assessed based on cfDNA copy number profiles and allele ratio of heterozygous single nucleotide polymorphisms (SNPs). All variants were manually inspected in the Integrative Genomics Viewer (IGV, v. 2.5.3).

Copy number variations (CNVs) were called using CNV Kit (v. 0.7.9)(10) and PureCN (v. 1.2.3)(11). Somatic focal amplifications were called if the median $\log_2$-ratio at a given gene exceeded control regions (defined as 3-8 Mb up- and downstream of gene start/end, respectively) by ≥ 0.5. Likewise, somatic focal deletions were called when the $\log_2$-ratio of control regions exceeded that of the gene by ≥ 0.3. All somatic amplifications and deletions

underwent manual curation in IGV (v. 2.5.3) and were considered real if supported by the SNP allele ratio. Homozygous deletions (log_2-ratio ≤-1) were defined as previously described (3).

Structural variants (SVs) were called using Svcaller (v. 1.0), SviCT (v. 1.0.1)(12), LUMPY (v. 0.3.0)(12), and SvABA (v. 1.1.0)(13). Variants called by only one caller were discarded, except when only called by Svcaller. All variants were manually inspected in IGV.

Variant impact was annotated using Ensembl Variant Effect Predictor (ensemble-vep v. 96.0)(14). Splice site alterations were further assessed for impact using multiple *in silico* tools (MaxEntScan, NNSplice)(15, 16).

mSINGS (v. 3.6)(17) was used for MSI analysis. Samples with a mSINGS fraction ≥ 0.2 were annotated as having MSI.

To estimate the fraction of cfDNA that is tumor derived (ctDNA%), tumor cell purity was calculated using somatic SNVs with moderate or high impact. For each sample, the SNV with the highest VAF was used. In cases with multiple driver SNVs with similar VAFs (+/- 2%), the median VAF was used. In cases with LOH of the SNV(s), purity was estimated as follows: tumor cell purity=2/(1/MAF+1), where MAF refers to mean allele frequency. In cases without LOH, purity was estimated as follows: tumor cell purity=2*MAF. Tumor cell purity was corrected for tumor ploidy to obtain ctDNA%, as follows: ctDNA% = tumor cell purity * tumor ploidy/(tumor cell purity * tumor ploidy + normal cell purity*normal ploidy), where normal purity = 1 - tumor cell purity and normal ploidy = 2. PureCN (v. 1.2.3)(11) was used to obtain tumor ploidies.

Clonal hematopoiesis

Likely clonal hematopoiesis (CH) variants were called based on the targeted sequencing of DNA from the buffy coat using GATK Haplotypecaller and Strelka2 Germline (5, 18), and annotated with Ensembl Variant Effect Predictor (14). CH variants were filtered out from both baseline and progression samples. Variants were filtered based on the following criteria: VAF ≤0.35, and affecting the protein-coding part of the gene, and within one of the 64 genes present in the CH compendium established by Pich et al. (19) with the addition of *BRCA1* and *BRCA2*, as CH variants in these genes have also been described (20, 21). All variants were manually inspected in IGV. Variants with obvious strand bias or low coverage were filtered out.

Exact VAFs of the final variants were calculated for both buffy coat and plasma samples using the reference and alternate read depths in IGV. VAFs in buffy coat samples were distributed similarly across patients (representative VAF distribution, Supplementary Figure 5) with major peaks around 0.50 and 1.00, denoting heterozygous and homozygous germline variants, respectively.

ddPCR analyses

All ddPCR assays comprised a single primer pair amplifying the target region, one mutation-specific probe (FAM-labeled), and one wild-type specific probe (HEX/VIC-labeled). ddPCR reactions were run in volumes of 22 µL, containing 9 µL cfDNA sample, 2 µL 20x primer-probe mix, and 11 µL 2x supermix for probes (Bio-Rad). Droplets were generated using the QX100 automated droplet generator system (Bio-Rad), read on a QX200 Droplet Reader (Bio-Rad), and analyzed using the Quantasoft software. Assay sequences and PCR conditions are specified in Supplementary Table 6.

cfDNA extracted from 2 mL plasma was split into 4 separate ddPCR reactions. As negative control, germline DNA extracted from buffy coats sampled at progression was diluted to 5 ng/µL and sonicated (Covaris) for 80 seconds yielding DNA fragments of ~300 bp. 2 µL sonicated germline DNA was diluted in 7 µL molecular-grade water and included in the ddPCR setup, alongside a non-template-control and a mutation-specific positive oligo (Supplementary Table 6). Thresholds for separating positive and negative ddPCR droplets were set manually using the amplitudes of sonicated germline DNA and the positive control. A cfDNA sample was defined as ctDNA-positive if at least three positive droplets were observed across the four ddPCR reactions. VAFs were calculated as the concentration of mutated amplicons divided by the total concentration of mutated and wild-type amplicons.

Clinical outcomes and statistical analysis

For clinical outcome analyses only pathogenic and likely pathogenic SNVs, SNVs annotated as high impact variants (22), amplifications, and homozygous deletions were included (i.e., structural variants and heterozygous deletions were excluded). The primary endpoint was PSA progression-free survival (PFS), defined as the time from first-line treatment initiation until time of PSA progression. If PSA initially decreased, progression was defined as an absolute

increase from PSA nadir of ≥2 ng/mL and ≥25%, as outlined in the recommendations from the Prostate Cancer Clinical Trials Working Group 3 (23). If PSA was stable or increased from baseline, PSA progression was defined as an absolute increase of ≥2 ng/mL and ≥25%, evaluated at earliest 12 weeks following treatment initiation (23). As a secondary endpoint, we used overall survival (OS) defined as the time from treatment initiation until death from any cause. PSA response was also defined as the proportion of patients achieving either a reduction of 50% (PSA50) or 90% (PSA90) from baseline, at earliest 12 weeks from start of treatment. Lastly, we defined primary resistance as treatment failure by 3 months.

Statistical analyses were conducted in R (v. 3.6.3), and in GraphPad Prism 9 (v. 9.5.1) with two-sided p-values <0.05 considered as statistically significant. In brief, Spearman's rank correlation was used to compare ctDNA% determined by different methods. Fischer's exact test was used to compare differences in the frequency of specific gene alterations between patients with primary resistance and those sensitive to treatment. For survival analyses, log-rank tests (along with Kaplan-Meier curves), and uni- and multivariate Cox regression were performed, including testing the proportional hazards assumption. The Benjamini-Hochberg (BH) method was used to adjust for multiple testing, as relevant.

References:

1. Norgaard M, Bjerre MT, Fredsoe J, Vang S, Jensen JB, De Laere B, et al. Prognostic Value of Low-Pass Whole Genome Sequencing of Circulating Tumor DNA in Metastatic Castration-Resistant Prostate Cancer. Clin Chem. 2023.
2. Reinert T, Scholer LV, Thomsen R, Tobiasen H, Vang S, Nordentoft I, et al. Analysis of circulating tumour DNA to monitor disease burden following colorectal cancer surgery. Gut. 2016;65(4):625-34.
3. Mayrhofer M, De Laere B, Whitington T, Van Oyen P, Ghysel C, Ampe J, et al. Cell-free DNA profiling of metastatic prostate cancer reveals microsatellite instability, structural rearrangements and clonal hematopoiesis. Genome Med. 2018;10(1):85.
4. David Benjamin TS, Kristian Cibulskis, Gad Getz, Chip Stewart, Lee Lichtenstein. Calling Somatic SNVs and Indels with Mutect2. bioRxiv. 2019.
5. Kim S, Scheffler K, Halpern AL, Bekritsky MA, Noh E, Kallberg M, et al. Strelka2: fast and accurate calling of germline and somatic variants. Nat Methods. 2018;15(8):591-4.
6. Lai Z, Markovets A, Ahdesmaki M, Chapman B, Hofmann O, McEwen R, et al. VarDict: a novel and versatile variant caller for next-generation sequencing in cancer research. Nucleic Acids Res. 2016;44(11):e108.
7. Koboldt DC, Zhang Q, Larson DE, Shen D, McLellan MD, Lin L, et al. VarScan 2: somatic mutation and copy number alteration discovery in cancer by exome sequencing. Genome Res. 2012;22(3):568-76.

8. Landrum MJ, Lee JM, Riley GR, Jang W, Rubinstein WS, Church DM, et al. ClinVar: public archive of relationships among sequence variation and human phenotype. Nucleic Acids Res. 2014;42(Database issue):D980-5.
9. Chakravarty D, Gao J, Phillips SM, Kundra R, Zhang H, Wang J, et al. OncoKB: A Precision Oncology Knowledge Base. JCO Precis Oncol. 2017;2017.
10. Talevich E, Shain AH, Botton T, Bastian BC. CNVkit: Genome-Wide Copy Number Detection and Visualization from Targeted DNA Sequencing. PLoS Comput Biol. 2016;12(4):e1004873.
11. Riester M, Singh AP, Brannon AR, Yu K, Campbell CD, Chiang DY, et al. PureCN: copy number calling and SNV classification using targeted short read sequencing. Source Code Biol Med. 2016;11:13.
12. Gawronski AR, Lin YY, McConeghy B, LeBihan S, Asghari H, Kockan C, et al. Structural variation and fusion detection using targeted sequencing data from circulating cell free DNA. Nucleic Acids Res. 2019;47(7):e38.
13. Wala JA, Bandopadhayay P, Greenwald NF, O'Rourke R, Sharpe T, Stewart C, et al. SvABA: genome-wide detection of structural variants and indels by local assembly. Genome Res. 2018;28(4):581-91.
14. McLaren W, Gil L, Hunt SE, Riat HS, Ritchie GR, Thormann A, et al. The Ensembl Variant Effect Predictor. Genome Biol. 2016;17(1):122.
15. Yeo G, Burge CB. Maximum entropy modeling of short sequence motifs with applications to RNA splicing signals. J Comput Biol. 2004;11(2-3):377-94.
16. Reese MG, Eeckman FH, Kulp D, Haussler D. Improved splice site detection in Genie. J Comput Biol. 1997;4(3):311-23.
17. Salipante SJ, Scroggins SM, Hampel HL, Turner EH, Pritchard CC. Microsatellite instability detection by next generation sequencing. Clin Chem. 2014;60(9):1192-9.
18. Poplin R R-RV, DePristo MA, Fennell TJ, Carneiro MO, Van der Auwera GA, Kling DE, Gauthier LD, Levy-Moonshine A, Roazen D, Shakir K, Thibault J, Chandran S, Whelan C, Lek M, Gabriel S, Daly MJ, Neale B, MacArthur DG, Banks E. Scaling accurate genetic variant discovery to tens of thousands of samples. bioRxiv. 2017.
19. Pich O, Reyes-Salazar I, Gonzalez-Perez A, Lopez-Bigas N. Discovering the drivers of clonal hematopoiesis. Nat Commun. 2022;13(1):4267.
20. Jensen K, Konnick EQ, Schweizer MT, Sokolova AO, Grivas P, Cheng HH, et al. Association of Clonal Hematopoiesis in DNA Repair Genes With Prostate Cancer Plasma Cell-free DNA Testing Interference. JAMA oncology. 2021;7(1):107-10.
21. Arends CM, Kopp K, Hablesreiter R, Estrada N, Christen F, Moll UM, et al. Dynamics of clonal hematopoiesis under DNA-damaging treatment in patients with ovarian cancer. Leukemia. 2024;38(6):1378-89.
22. McLaren W, Gil L, Hunt SE, Riat HS, Ritchie GRS, Thormann A, et al. The Ensembl Variant Effect Predictor. Genome Biology. 2016;17(1):122.
23. Scher HI, Morris MJ, Stadler WM, Higano C, Basch E, Fizazi K, et al. Trial Design and Objectives for Castration-Resistant Prostate Cancer: Updated Recommendations From the Prostate Cancer Clinical Trials Working Group 3. Journal of clinical oncology : official journal of the American Society of Clinical Oncology. 2016;34(12):1402-18.
