# Supplementary material for: Deep targeted sequencing of circulating tumor DNA to inform treatment in patients with metastatic castration-resistant prostate cancer
Source: J Exp Clin Cancer Res. 2025 Apr 14;44:120. doi: 10.1186/s13046-025-03356-0 (PMC11998381; doi:10.1186/s13046-025-03356-0)
Supplement: Supplementary file 1 — Supplementary Material 1. [file 13046_2025_3356_MOESM1_ESM.zip › Supplementary Materials/Supplementary Figure Legends.pdf]

Supplementary Figure 1: Clinical outcomes stratified by ctDNA fraction. Kaplan Meier plots of patients with ctDNA% <3% enrolled during the same study period as the cohort herein, compared to those with ctDNA% ≥ 3% (current study cohort), using a) PSA PFS and b) OS as endpoints. P-values in Kaplan-Meier plots based on log-rank test.

Supplementary Figure 2: Overview of germline alterations. Oncoplot of germline variants in the patient population. All variants identified were SNVs. ACMG classification is indicated. Patients are ordered according to months to PSA progression (top barplot, red line indicates cut-off for primary resistance defined as treatment failure within the first three months). ctDNA fraction as determined by ichorCNA is shown in the bottom barplot.

Supplementary Figure 3: Overview of alterations at baseline compared to progression on first-line treatment. Patients (n=18) are ordered according to months to PSA progression (top barplot, red line indicates cut-off for primary resistance). ctDNA fraction as determined by ichorCNA is shown in the bottom barplot. (AMP, amplification; HET-DEL, heterozygous deletion; HOM-DEL, homozygous deletion; MSI, microsatellite instability; SNV, small nucleotide variant; SV, structural variant)

Supplementary Figure 4: Patient-specific ddPCR assays showing the longitudinal dynamics of specific variants detected in ctDNA at both baseline and progression. Changes in PSA shown, as well as VAF for the patient-specific alteration, based on ddPCR. Open circles represent time points where the variant was not detected. Shaded region indicates time from initial PSA progression to treatment discontinuation.

Supplementary Figure 5: Representative variant allele frequency distribution from one patient (DNA extracted from buffy coat, baseline).
