# Supplementary material for: Deep targeted sequencing of circulating tumor DNA to inform treatment in patients with metastatic castration-resistant prostate cancer
Source: J Exp Clin Cancer Res. 2025 Apr 14;44:120. doi: 10.1186/s13046-025-03356-0 (PMC11998381; doi:10.1186/s13046-025-03356-0)
Supplement: Supplementary file 1 — Supplementary Material 1. [file 13046_2025_3356_MOESM1_ESM.zip › Supplementary Materials/Supplementary Table 3.pdf]

Supplementary Table 3: Likely CH and CHIP SNVs detected in baseline and progression samples. (CH, clonal hematopoiesis; CHIP, clonal hematopoiesis of indeterminate potential (i.e. pathogenic CH variants with VAF>2%), VAF, variant allele frequency) *Only baseline sample available.

**Patient**	**Sampling time**	**Gene**	**Variant**		**CH/CHIP**	**VAF buffy coat**	**VAF plasma**	**ctDNA% plasma**
**29***	Baseline	DNMT3A	2398G>A	Gly800Ser	CH	8.4	7.9	26.7%
**31**	Baseline	CHEK2	886G>A	Asp296Asn	CH	9.5	4.1	14.5%
**40**	Baseline	BRCA2	1159G>A	Val387Ile	CH	8.2	7.5	10.1%
	Progression				CH	9.1	3.4	46.2%
**38***	Baseline	KMT2C	1131A>T	Pro377=	CH	26.4	22.7	9.1%
**49***	Baseline	TP53	701A>G	Tyr234Cys	CHIP	9.2	9.6	3.4%
**36**	Progression	SF3B1	1998G>T	Lys666Asn	CHIP	9.7	11.2	9.5%
**33**	Progression	KMT2C	1131A>T	Pro377=	CH	18.5	21.6	6.8%
