# Supplementary material for: Deep targeted sequencing of circulating tumor DNA to inform treatment in patients with metastatic castration-resistant prostate cancer
Source: J Exp Clin Cancer Res. 2025 Apr 14;44:120. doi: 10.1186/s13046-025-03356-0 (PMC11998381; doi:10.1186/s13046-025-03356-0)
Supplement: Supplementary file 1 — Supplementary Material 1. [file 13046_2025_3356_MOESM1_ESM.zip › Supplementary Materials/Supplementary Table 5.pdf]

Supplementary Table 5: Univariate and multivariate Cox regression for Annala et al. cohort using PSA PFS and OS as endpoints. Multivariate analyses corrected for ctDNA fraction. Only pathogenic and likely pathogenic SNVs, SNVs annotated as high impact variants, amplifications, and homozygous deletions were included in these analyses (i.e., structural variants and heterozygous deletions were excluded). (Alt., altered; BH, Benjamini-Hochberg; CI, confidence interval; HR, hazard ratio; OS, overall survival; PFS, progression-free survival; Wt, wild-type). Analyses that failed the cox proportionality test are marked as NA.

Alteration	Number of patients (n=202)		Univariate analyses				Multivariate analyses			
			PSA PFS		OS		PSA PFS		OS	
	Alt	Wt	HR (95% CI)	p-value (BH adj.)	HR (95% CI)	p-value (BH adj.)	HR (95% CI)	p-value (BH adj.)	HR (95% CI)	p-value (BH adj.)
PTEN	14	188	2.09 (1.15-3.81)	0.015 (0.038)	3.02 (1.48-6.16)	0.0024 (0.011)	1.53 (0.84-2.8)	0.165 (0.28)	1.97 (0.96-4.1)	0.064 (0.13)
Cell cycle (CCND1,CDKN1B, CDKN2A, RB1)	16	186	1.44 (0.81-2.56)	0.22 (0.28)	1.61 (0.76-3.40)	0.22 (0.28)	NA	NA	NA	NA
Chromatin modulators (ARID1A, CHD1)	6	196	3.47 (1.52-7.93)	0.0032 (0.011)	4.37 (1.87-10.21)	0.00065 (0.0065)	1.55 (0.65-3.68)	0.32 (0.34)	1.54 (0.63-3.75)	0.34 (0.34)
